# Antitumor activity of Cetuximab in combination with Ixabepilone on triple negative breast cancer stem cells

**DOI:** 10.1186/s13058-015-0662-4

**Published:** 2016-01-12

**Authors:** Tomonori Tanei, Dong Soon Choi, Angel A. Rodriguez, Diana Hwang Liang, Lacey Dobrolecki, Madhumita Ghosh, Melissa D. Landis, Jenny C. Chang

**Affiliations:** 1 Methodist Cancer Center, Houston Methodist Hospital, 6445 Main Street, P21-34, Houston, TX 77030 USA; 2 Department of Surgery, Houston Methodist Hospital, Houston, TX 77030 USA

## Abstract

**Background:**

Developing novel strategies against treatment-resistant triple negative breast cancer (TNBC) cells remains a significant challenge. The ErbB family, including epidermal growth factor receptor (EGFR), plays key roles in metastasis, tumorigenesis, cell proliferation, and drug resistance. Recently, these characteristics have been linked to a small subpopulation of cells classified as cancer stem cells (CSC) which are believed to be responsible for tumor initiation and maintenance. Ixabepilone is a new generation microtubule-stabilizing agent, which has been expected to be more efficacious than conventional taxanes. Here we aim to investigate whether the EGFR monoclonal antibody Cetuximab, in combination with Ixabepilone, is more effective in eliminating CSC populations compared to chemotherapy alone in TNBC.

**Methods:**

Representative TNBC cell lines (MDA-MB-231 and SUM159) were used to evaluate breast CSC populations. We used fluorescence-activated cell sorter analysis (CD44^+^ and CD24^-/low^, or Aldefluor^+^) and a self-renewal assay called mammosphere formation efficiency (MSFE) to measure CSC population size after treatment with Cetuximab, or Cetuximab plus Ixabepilone *in vitro*.

**Results:**

Although there was no significant decrease in cell viability, Cetuximab reduced MSFE and the CSC population in breast cancer cells *in vitro* and *in vivo* through inhibition of autophagy. Also, SUM159 and MDA-MB-231 orthotopic tumors demonstrated partial response to Centuximab or Ixabepilone monotherapy; however, the effect of the combination treatment was significant only in SUM159 tumors (p <0.0001), when compared to Ixabepilone alone.

**Conclusions:**

Overall, our findings demonstrate that EGFR-targeted therapy by Cetuximab effectively reduces the CSC population in TNBC tumors. However, combination therapy with Ixabepilone may be effective only in a small subset of TNBCs, warranting further investigation of alternative approaches to target multiple pathways for TNBC treatment.

**Electronic supplementary material:**

The online version of this article (doi:10.1186/s13058-015-0662-4) contains supplementary material, which is available to authorized users.

## Background

Triple-negative breast cancer (TNBC), which accounts for 20 % of all breast cancers, is characterized by the absence of estrogen receptor (ER), progesterone receptor (PR), and human epidermal growth factor receptor 2 (HER2) expression. They are histologically high grade, aggressive, and lethal tumor types that lack targeted therapeutic options. Patients with TNBC are associated with relatively poor prognosis and are at a significant risk of relapse and frequent metastases [1, 2]. Triple-negative and basal-like breast cancers display a similar profile of cell-surface markers of breast cancer stem cells (CSCs) [3]. CSCs are defined as rare tumor cells that are capable of self-renewal and give rise to multipotent progenitor cells, which ultimately differentiate into all cell types within the tumor [4–6]. CSCs have been identified by cell sorting technologies using various surface markers in acute myeloid leukemia and solid tumors, including breast tumors [7]. Studying tumorigenic cells separated in vitro, from malignant human breast cancer-derived pleural effusions, Al Hajj and colleagues isolated a cell population characterized by high CD44 expression and low or undetectable levels of CD24 (CD44^+^/CD24^−/low^). These cells had classic features of bona fide stem cells, including the capacity for self-renewal and generation of heterogeneous progeny [8]. This subpopulation can form mammospheres in vitro and were shown to be enriched for tumorigenic cells by their ability to form xenograft tumors in immunocompromised mice [8]. Ginestier et al. demonstrated that aldehyde dehydrogenase 1 is an alternative marker for breast CSCs [8]. We have recently shown that CD44^+^/CD24^−/low^ and ALDH^+^ phenotypes reflect different epithelial-mesenchymal transition states in CSCs [9]. Identification of breast CSCs from tumor samples or breast cancer cell lines has been based mainly on CD44^+^/CD24^−/low^ or ALDH^+^ phenotypes. We have previously reported that breast CSCs are a subpopulation of cells within the primary tumor responsible for tumor initiation and metastases, and are associated with resistance to chemotherapy in human breast cancers following neoadjuvant chemotherapy [10]. In addition, it has been shown that epidermal growth factor receptor (EGFR) signaling may be required for cancer self-renewal [11]. EGFR is more commonly overexpressed in TNBC than in other breast cancer subtypes [12, 13]. Also, TNBC can be classified as basal type cancer defined by EGFR and cytokeratin 5/6 staining.

Ixabepilone is a new-generation microtubule-stabilizing agent and has more efficacious anti-tumor effects than taxanes [14, 15]. It is an analog of epothilone B, a naturally occurring microtubule stabilizer with very high cytotoxic activities against a wide range of tumor types, including drug-resistant tumors. For example, anthracycline- and taxane-resistant metastatic breast cancers (MBCs) are known to be highly sensitive to Ixabepilone as a single agent or in combination with Capecitabine [13]. Importantly, significant anticancer activity was seen in ER, PR, HER2 negative TNBC patients with MBC. This is consistent with the preclinical activity of Ixabepilone against human cancer cell lines resistant to taxanes and other agents [16].

Combination therapy is a mainstay of anticancer treatment, with optimal combinations producing synergistic antitumor responses. This is achieved by combining agents with established safety profiles and non-overlapping mechanisms of action. Thus, this study seeks to evaluate the combination therapy of combining Cetuximab and Ixabepilone, which might be more effective at targeting cancer stem cells than other antitubulins, as a possible way to increase antitumor activity. In the present study, we investigated whether the breast CSC population enriched for tumorigenic CD44^+^CD24^−/low^ cells could be eradicated when treated with Cetuximab and Ixabepilone, as opposed to chemotherapy alone, in TNBC xenografts. Our findings suggest that Ixabepilone produces therapeutic synergism with Cetuximab only in a small subset of TNBCs and provides additional evidence that clinical trials using Cetuximab in combination with Ixabepilone should be applied with caution.

## Methods

### Cell proliferation and viability measurements (WST-1 assay)

TNBC cell lines MDA-MB-231 and SUM159 were purchased from American Type Culture Collection (ATCC), Manassas, VA, USA, and from Asterand, Detroit, MI, USA, These cell lines were chosen based on their high expression of epithelial-mesenchymal transition markers (EMT), metastatic properties, and percentage of CD44+ /CD24- cells. Both cell lines were grown in DMEM (Invitrogen, Carlsbad, CA) with 10 % fetal bovine serum (Cellgro, Manassas, VA) and 1 % penicillin/streptomycin. WST-1 assay was performed using Premixed WST-1 Cell Proliferation Reagent (Clontech, Mountain View, CA) according to the manufacturer’s protocol to access cell viability. Cells were seeded into 96-well tissue culture plates at the concentration of 2,000 cells/well and incubated at different concentrations of Cetuximab (Erbitux, Bristol Myers Squibb, NJ) (0.001–100 μg/ml), Cetuximab (1 nM–100 uM) or in combination with Ixabepilone (Ixampra, Bristol Myers Squibb, NJ) for 72 h. Cell viability was analyzed indirectly by measuring formazan formation by mitochondrial dehydrogenases in viable cells. The absorbance was measured using a multiwall scanning spectrophotometer (Infinite M200 pro, Tecan, Switzerland) at 440 nm (measurement wavelength) and 600 nm (reference wavelength).

### Fluorescence-activated cell sorting (FACS) for CD44^+^CD24^−/low^ cell isolation

To analyze CD44^+^/CD24^-/low^ cells, approximately 10^6^ cells were resuspended in Hank’s balanced salt solution (HBSS) supplemented with 2 % fetal bovine serum, mixed, and incubated for 15 minutes at 4 °C with mouse monoclonal anti-CD44-APC (0.6 μg/100 μl/test) and anti-CD24–PE (4 μg/100 μl /test) (BD Pharmingen, San Jose, CA, USA) according to the manufacturer’s instructions. The cells were then washed and resuspended in HBSS (Invitrogen, Carlsbad, CA, USA) supplemented with 1 M HEPES. Propidium iodide (PI) was added to exclude dead cells before FACS analysis using BD LSRII flow cytometry (BD Bioscience, San Jose, CA, USA). Only live and single cell populations were gated out for population analysis using a PI gate and side and forward scatter gates. Negative and positive controls were stained with antibodies of isotype control, PE (phycoerythrin) positive control, APC (allophycocyanin) positive control, or fluorescein isothiocyanate (FITC) positive control. All experiments were repeated three times.

### Aldefluor (ALDF) assay

The Aldefluor assay was performed as described by the manufacturer (Stem cell technology, BC, Canada). Briefly, 5 × 10^5^ cells were incubated for 45 minutes at 37 °C with ALDF cocktail (ALDF reagent; 2.5 μl, ALDF Buffer; 500 μl/test). After washing the ALDF buffer, the cells were rinsed and resuspended in HBSS + 1 M HEPES. For xenograft tumors, cells were additionally stained with anti-H2Kd-PE (2.5 μl/100 μl/test) antibodies on ice for 15 minutes to exclude mouse components. We used cells stained with DEAB cocktail (DEAB reagent; 5 μl ALDF reagent; 2.5 μl ALDF buffer; 500 μl/test) as negative controls.

### Mammosphere-forming assay

Mammosphere-forming efficiency (MSFE) was measured by counting spheres as previously described [17, 18]. In brief, cancer cells (5,000 cells/ml) were cultured for 72 h in ultralow attachment 24-well plates (Corning Costar Co., MA, USA) with the mammosphere culture medium Plus (MEGM+, Lonza, Switzerland) containing 20 ng/ml of bFGF (Invitrogen, Carlsbad, CA, USA), 20 ng/ml of EGF (Invitrogen), 4 μg/ml of heparin (StemCell Technologies, BC, Canada), and B27 supplement (Invitrogen). Cells were treated at the time of cell seeding with Cetuximab, Ixabepilone, or the combination at the indicated concentrations. Mammospheres were counted using the Gel Counter (Optronix, UK) and GelCount software. To calculate the MSFE, the following formula was used:

$$ \mathrm{MSFE}\ \left(\%\right) = \left(\mathrm{Total}\ \mathrm{number}\ \mathrm{of}\ \mathrm{spheres}\kern0.75em \times 100\right)/\mathrm{Total}\ \mathrm{number}\ \mathrm{cells}\ \mathrm{seeded} $$

Each experiment was repeated at least three times with six replicates for each treatment group.

### Animal experiments

All animal protocols were reviewed and approved by the Animal Protocol Review Committee at Houston Methodist Research Institute (Houston, TX, USA). MDA-MB-231 or SUM159 cells (2 × 10^6^ cells/100 ml) were injected orthotopically in female immunocompromised severe combined immunodeficiency (SCID)/Beige mice (Charles River Laboratories, Wilmington, MA, USA). After tumors reached 5–8 mm in diameter, mice were randomly grouped based on tumor sizes and injected intravenously with (A) control, intravenous (i.v.) PBS, (B) Cetuximab, i.v. (4 mg/kg/weekly) (C) Ixabepilone, intraperitoneal (i.p.) (10 mg/kg/weekly), (D) Cetuximab, i.v. + Ixabepilone, i.p. (equivalent dose/weekly). Tumor diameters and body weights were recorded twice per week, and mice were euthanized at the end of treatment or when the recurrent tumor size reached the basal level. The tumors were then excised, minced, and digested in mammary epithelial growth medium (MEGM) with 200–250 U/ml of Collagenase type III (Worthington, NJ) and 0.8 units/ml of Dispase (Worthington, NJ) in a shaking incubator at 37 °C for 1.5 h. The samples were filtered, subjected to hypotonic shock (9 ml of sterile H_2_O for 10 seconds followed with 1 ml of 10 × HBSS) to lyse red blood cells, washed with HBSS, and finally re-filtered for single cells using 40-μm pore filters. To analyze MSFE, cells (20,000 cells/ml) were plated in the ultralow attachment, as described above, for 2 weeks.

### Western blot assay

Western blotting was performed as previously described [17]. Briefly, cells were lysed with 1X Cell Lysis Buffer (Cell Signaling Tech., Danvers, MA, USA). After measuring the protein concentration using a Pierce BCA protein assay kit (Thermo Fisher Scientific Inc, Waltham, MA, USA), a total of 300 μg of protein extract from each sample was mixed with 4 × sample buffer (Bio-Rad, Hercules, CA, USA) containing 5 % β-mercaptoethanol (Sigma, St. Louis, MO, USA). Samples (30 μg/well) were loaded onto a 4–20 % SDS-PAGE gel and transferred to nitrocellulose membranes (Bio-Rad). All antibodies were purchased from Cell Signaling Technology: phospho-EGF receptor (Tyr1068) antibody, EGF receptor antibody (1 F4), phospho-AKT (Ser473) antibody, AKT antibody, phospho-extracellular signal-regulated kinase (ERK)1/2 antibody, ERK1/2 antibody, p62, LC3b, and β-Actin.

### Statistical analyses

All quantitative parameters are presented as mean with standard deviation, and all results are expressed as mean ± standard error of the mean (SEM) for in vivo experiments. In vitro (WST-1 assay, flow cytometry, or mammosphere-forming efficiency) results were analyzed using Student’s *t* test, and the Mann–Whitney *U* test for unpaired samples. In vivo data were statistically evaluated by means of two-way analysis of variance (ANOVA). Significant treatment effects were subsequently delineated by using Dunnett’s post hoc test. Statistical significance was assumed for *p* <0.05.

## Results

### Cell viability of triple-negative cell lines treated with Cetuximab and Ixabepilone

**Fig. 1:**
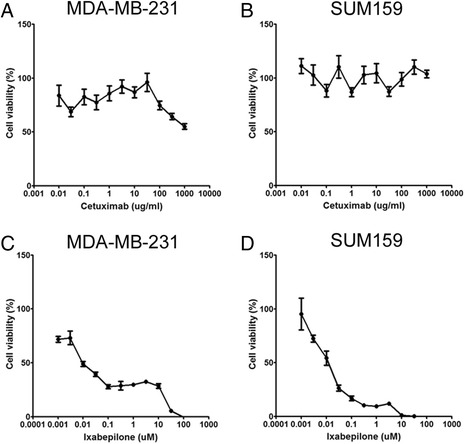
The effect of Ixabepilone or Cetuximab on cell viability of triple-negative breast cancer cell lines with WST-1. WST-1 proliferation assay of MDA-MB-231 cells and SUM159 cells treated with Cetuximab (MDA-MB-231 cells (**a**) SUM159 cells (**b**)), or Ixabepilone (MDA-MB-231 cells (**c**), SUM159 cells (**d**)). The data for each cell line are mean ± standard deviation obtained from three independent experiments

Cell viability was analyzed using the WST-1 assays of Cetuximab or Ixabepilone in the TNBC cell lines MDA-MB-231 and SUM159 (Fig. 1). The viability of these cells did not change with increasing doses of Cetuximab (Fig. 1a and b). On the other hand, both cell lines were sensitive to Ixabepilone in a statistically significant dose-dependent manner (*p* <0.01) (Fig. 1c and d). The inhibitory concentration (IC)_50_ was 10 ± 3 nM for MDA-MB-231 cells and 8 ± 2 nM for SUM159 cells. We found that Ixabepilone was the main contributor to cytotoxicity in the combination regimen of Cetuximab and Ixabepilone (data not shown).

### Breast CSCs are sensitive to Cetuximab

**Fig. 2:**
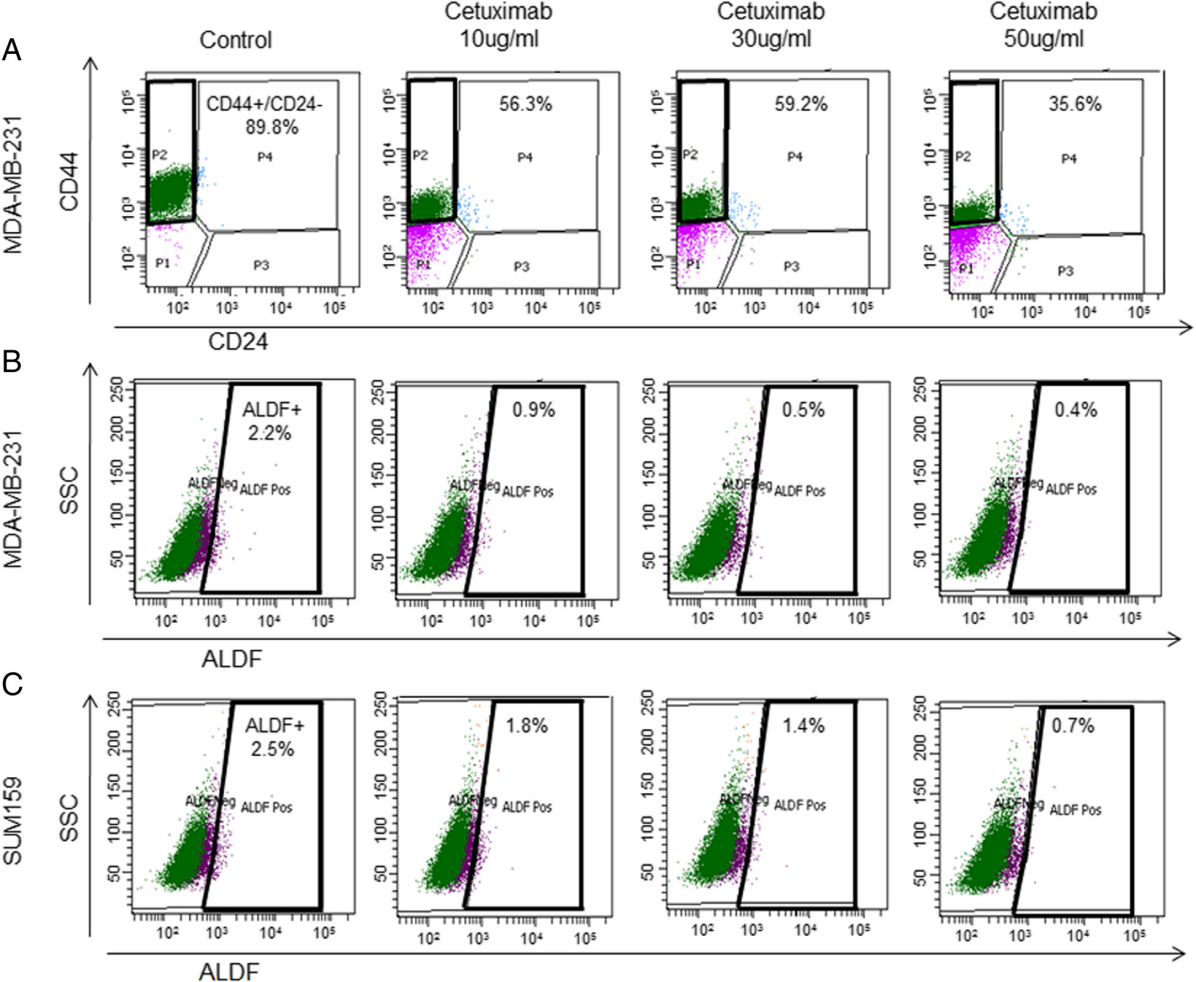
FACS analysis for double staining of CD44^+^/CD24^-/low^ and Aldefluor (*ALDF*) in MDA-MB-231, or Aldefluor in SUM159. Representative results of FACS analysis identified by CD44^+^/CD24^-/low^ in MDA-MB-231 (**a**), Aldefluor + in MDA-MB-231 (**b**), and Aldefluor + in SUM159 (**c**) treated with different doses of Cetuximab (10 μg/ml, 30 μg/ml, or 50 μg/ml) for 3 days. Proportion (%) of CD44^+^/CD24^-/low^ or Aldefluor + cells were determined and represented by the average of FACS analysis performed in triplicate

We evaluated the effects of Cetuximab on the CSC population by analyzing CD44^+^/CD24^-/low^, or ALDF^+^ populations using FACS. The tested doses of Cetuximab were 10, 30, and 50 μg/ml. After treatment with Cetuximab for 3 days, a dose-dependent reduction in the CD44^+^/CD24^-/low^ population of MDA-MB-231 cells was observed and the reduction was highest at a dose of 50 μg/ml, from 89.8 % to 35.8 % (*p* <0.01) (Fig. 2a). Cetuximab also reduced the ALDF^+^ population dose-dependently, with a maximum decrease from 2.2 to 0.4 % (*p* <0.01) at 50 μg/ml of Cetuximab (Fig. 2b). Similarly, the highest concentration of Cetuximab reduced the ALDF+ population from 2.5 to 0.7 % (*p* <0.01) in SUM159 cells in a dose-dependent manner (Fig. 2c).

### Cetuximab inhibits clonogenicity of TNBC cells

**Fig. 3:**
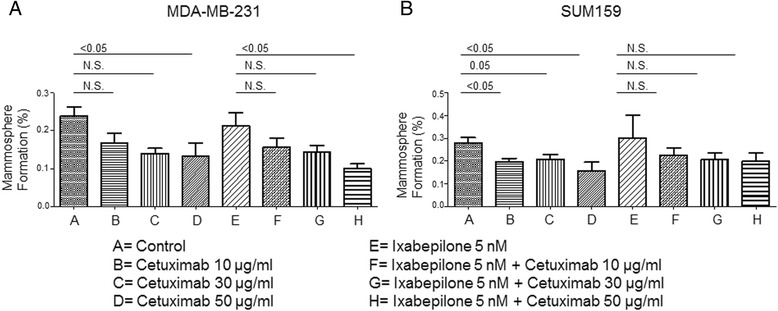
Mammosphere-forming efficiency of MDA-MB-231 and SUM159 cells treated with Cetuximab +/- Ixabepilone. Mammosphere formation (%) of MDA-MB-231 (**a**) and SUM159 (**b**) cells was compared between control (no treatment) and Cetuximab treatment at concentrations of 10 μg/ml, 30 μg/ml, 50 μg/ml or between Ixabepilone (5 nM) and Ixabepilone (5 nM) + Cetuximab treatment at 10 μg/ml, 30 μg/ml, 50 μg/ml. The Mann–Whitney *U* test was used to determine the *p* values. Data represent the mean of three independent experiments. *N.S.* not significant

Mammosphere-forming assays were performed to evaluate the effects of Cetuximab alone or in combination with Ixabepilone on MSFE of TNBC cells by measuring MSFE. The results showed that Cetuximab alone decreased MSFE in MDA-MB-231 cells from 0.24 to 0.13 % (*p* <0.05) and in SUM159 cells from 0.28 % to 0.16 %, (*p* <0.05) (Fig. 3a and b). Ixabepilone monotherapy had no effect on MSFE in either cell line. We also found that the combination therapy significantly reduced the MSFE of MDA-MB-231 cells at the 50 μg/ml dose of Cetuximab (the mean MSFE at Ixabepilone 5 nM of 0.21 % versus Ixabepilone 5 nM + Cetuximab 50 μg/ml of 0.10 %, *p* <0.05) (Fig. 3a). The combination treatment resulted in a reduction in MSFE for SUM159 cells, but the change was not statistically significant (mean at Ixabepilone 5 nM of 0.30 % versus Ixabepilone 5 nM + Cetuximab 50 μg/ml of 0.20 %, *p* >0.05) (Fig. 3b). These results suggest that the EGFR signaling pathway may be critical for clonal expansion of CSCs.

### Tumor growth of TNBC xenografts with Cetuximab treatment

**Fig. 4:**
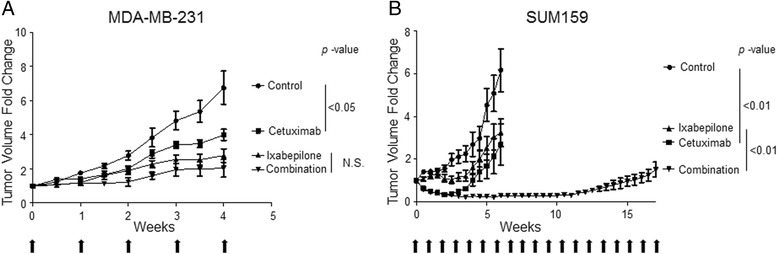
MDA-MB-231 and SUM159 tumor models of xenograft mice treated with Cetuximab, Ixabepilone, and combination. Means of the percentage in tumor volume of control (intravenous (i.v.), PBS), Cetuximab (i.v., 4 mg/kg/weekly), Ixabepilone (intraperitoneal (i.p.), 10 mg/kg/weekly), or combination (Cetuximab, i.v + Ixabepilone, i.p (equivalent dose/weekly))-treated MDA-MB231 (**a**) or SUM159 (**b**) tumors in SCID/Beige mice were plotted as a function of time. Two-way analysis of variance with Bonferroni post-hoc test was used to determine significant differences among groups and *p* <0.05 was considered to be statistically significant

We established human breast cancer cell xenografts in immunocompromised SCID/Beige mice using MDA-MB-231 or SUM159, and divided them into four groups (control, Cetuximab, Ixabepilone, and combination). As shown in Fig. 4, Cetuximab therapy alone was effective in both MDA-MB-231 (tumor doubling time, TDT = 14 ± 3 days, *p* <0.05) and SUM159 (TDT = 30 ± 10 days, *p* <0.05) xenografts compared to control (TDT = 16 ± 3 days). In addition, the combined therapy induced a dramatic reduction in the tumor growth of SUM159 xenografts (TDT = 110 ± 25 days, *p* <0.001) compared to Ixabepilone alone (TDT = 25 ± 8 days) (Fig. 4b). The combination treatment in MDA-MB-231 xenografts caused a decrease in tumor growth, but was not statistically significant (Fig. 4a). Also, the combination treatment was not toxic, and the weight curves of the combined treatment groups were similar to other groups in the xenografts (data not shown). These results suggest that Cetuximab is effective against CSCs, and treating TNBC xenografts with the combination therapy did not result in additive toxicity.

### Effects of Cetuximab treatment on breast CSCs in TNBC xenografts

**Fig. 5:**
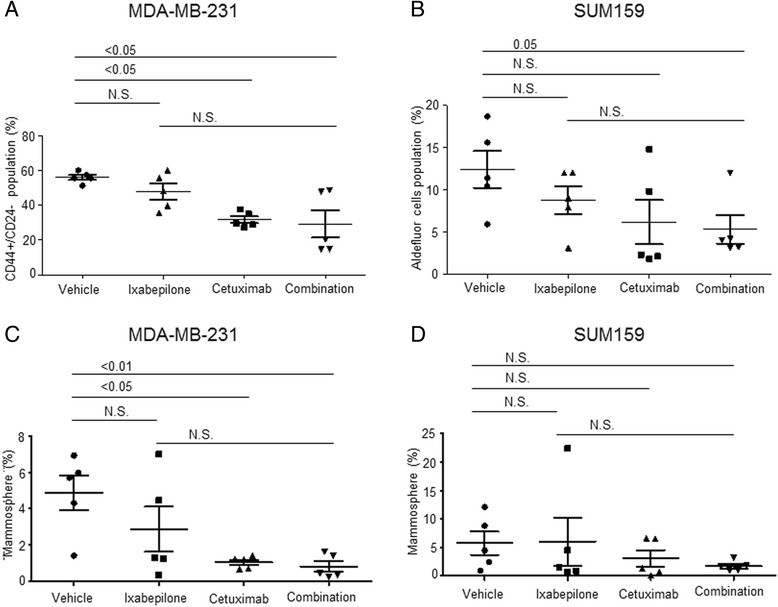
FACS analysis and mammosphere-forming efficiency in MDA-MB-231 or SUM159 xenografts. Representative results of CD44^+^/CD24^-/low^ population (%) in MDA-MB-231 xenografts (**a**), Aldefluor + population (%) in SUM159 xenografts (**b**), mammosphere formation (%) in MDA-MB-231 xenografts (**c**), or mammosphere formation (%) in SUM159 xenografts (**d**). CD44^+^/CD24^-/low^ population (%) and mammosphere formation (%) of MDA-MB-231 xenografts were reduced by Cetuximab alone or combination treatment. Aldefluor + population (%) in SUM159 xenografts decreased by combination treatment when compared to control. CD44+/CD24- cells (%) in SUM159 xenografts were extremely low in proportion and are not represented in the figure. *N.S.* not significant

To evaluate the effect of Cetuximab, Ixabepilone, and combined treatment on breast CSCs in MDA-MB-231 and SUM159 xenografts, we performed both FACS analysis and MSFE assays of xenograft tumor specimens taken after treatment. In MDA-MB-231 xenograft tumors, the mean percentage of CD44^+^/CD24^-/low^ cells decreased after treatment with Cetuximab (31.8 %, *p* <0.05) or the combination treatment (29.3 %, *p* <0.05) compared to control (56.1 %) (Fig. 5a). Of note, ALDF + cells were not identified in most of the MDA-MB-231 xenograft tumors (Fig. 5e). The MSFE of MDA-MB231 xenografts decreased after Cetuximab treatment (mean = 2.9 %, *p* <0.01) or combination (mean = 0.8 %, *p* <0.05) compared to control (mean = 4.9 %) (Fig. 5c). On the other hand, the mean ALDF + percentage decreased by the combination treatment in SUM159 xenograft tumors (5.3 %, *p* = 0.05) compared to control (12.4 %) (Fig. 5b). However, CD44^+^/CD24^-/low^ cells were not identified in most SUM159 xenograft tumors. Cetuximab treatment decreased the MSFE of SUM159 xenografts, but not to statistical significance (Fig. 5d). These results show that Cetuximab has the ability to decrease CSC populations in TNBC xenograft tumors.

### Cetuximab-treated tumors have decreased autophagy (LC3b, p62 and autophagosomes)

To determine whether Cetuximab targets the EGFR signaling pathway and autophagy, we performed immunoblotting to determine the expression levels of EGFR, phospho-EGFR, LC3b-I and II, and p62 against the effects of Cetuximab, Ixabepilone, and the combination treatment in MDA-MB-231 and SUM159 cells (Additional file 1: Figure S1). Western blot analysis revealed that Ixabepilone treatment increased the expression of several proteins including EGFR, phospho-EGFR, phospho-AKT(473), and phospho-ERK1/2 in both cell lines. Cetuximab reduced phospho-AKT(473) and phospho-ERK1/2, which are downstream of EGFR in the signaling pathway. In addition, Cetuximab and especially the combination treatment downregulated the autophagy markers p62 and LC3b-II in MDA-MB-231 and SUM159 cells compared to control or Ixabepilone. Although Ixabepilone increased p62 expression, Ixabepilone either reduced or had no effect on the basal levels of LC3b-II in MDA-MB-231 and SUM159 cells, respectively. These results suggest that Ixabepilone upregulated autophagy. Together, these findings provide strong evidence that Cetuximab inhibits the Akt and Erk1/2 pathways, resulting in downregulation of p62 and LC3b. Moreover, the combination treatment decreased autophagy by inhibiting Ixabeplione-induced EFGR activation.

## Discussion

Tumorigenic CSCs are intrinsically resistant to conventional chemotherapy and have unique properties including enhanced self-renewal and increased propensity for tumor formation [10, 19]. CSCs have been identified in various tumors including those of the breast, and they are particularly enriched in the basal-like and claudin-low subtypes of breast cancer. Cell lines representing the basal-like subtype (SUM159) and claudin-low subtype (MDA-MB-231) of breast cancer were utilized to study the anti-CSC effects of Cetuximab in combination with Ixabepilone. Our results indicate that Cetuximab alone or in combination with Ixabepilone significantly inhibited tumor growth and reduced CD44^+^/CD24^-/low^ CSC population in vitro and in vivo. However, the efficacy of the combination therapy varied in the two in vivo models compared to Ixabepilone monotherapy in that the effects of the combination were far greater in SUM159 tumors than in MDA-MB-231 tumors. Trédan et al. [20] recently reported that the clinical effectiveness of the Cetuximab and Ixabepilone combination was similar to that of Ixabepilone monotherapy in a first-line treatment of locally advanced or metastatic TNBC. Similarly, the results of two additional independent randomized phase II clinical trials, evaluating the effects of Cetuximab in combination with either cisplatin [21] or carboplatin [22], were similar to the clinical trial with Cetuximab and Ixabepilone. Carley et al. [22] separated patients into two groups, non-responders and responders, to the combination therapy by analyzing signaling signatures in post-treatment specimens. This revealed that low KRAS-amplicon expression was associated with a better response to the combination therapy of Cetuximab and carboplatin. In agreement with the results of the previous clinical trials, our data using two animal models reflect the disputed sensitivity of TNBC to Cetuximab in combination with chemotherapy. According to the Pietenpol TNBC classification [23], both cell lines are classified as mesenchymal-like. However, SUM159 cells have an *H-Ras* mutation and exhibit anaplastic carcinoma histology while MDA-MB-231 cells have a *K-Ras* mutation and invasive ductal carcinoma histology. These features may have contributed to the minimal response observed in MDA-MB-231 tumor models with the combination treatment. Additionally, differences in sensitivity to Ixabepilone may be another possible contributor to the bifurcated drug response with combination therapy, as our data indicate that SUM159 cells are more sensitive to Ixabepilone than MDA-MB-231 cells both in vitro and in vivo.

It was intriguing to observe that Cetuximab was not cytotoxic, but reduced CSCs in both cell lines in vitro and in vivo. This result is in agreement with previous reports that Cetuximab has little impact on cell viability [24, 25]. We speculate that the EGFR signaling pathway may be critical to maintain the stemness of CSCs, and that loss of EGFR signaling may induce differentiation of the CSCs to a non-CSC population. This notion is supported by data from Harrison et al. [26], Yang et al. [27], and Gillian et al. [28], showing that EGFR signaling is critical for CSC stemness in human and murine breast CSCs. Previously, we reported that the treatment of patients with HER2-positive tumors using lapatinib (an EGFR/HER2 inhibitor) led to a statistically non-significant decrease in the percentage of CD44^+^/CD24^-/low^ cells, and also a significant decrease in MSFE [29]. Despite effective targeting of the CSC population, our data suggested that inhibiting EGFR by Cetuximab in combination with Ixabepilone did not have significant anti-cancer effects in TNBC tumors. We hypothesize that either tumor heterogeneity or the development of compensatory mechanisms may have played a role in the minimal response to the Cetuximab and Ixabepilone combination. Supporting this hypothesis, Jacobsen et al. [30] recently showed that targeting EGFR can trigger compensatory activation of other ErbB family receptors, HER2 and/or HER3, and that simultaneous inhibition of EGFR, ErbB2 and ErbB3 effectively overcame tumor heterogeneity and plasticity.

Previously, we reported that the CSC population increases after chemotherapy in both clinical and preclinical settings [17, 29, 31], and that autophagy is the underlying mechanism for the survival and maintenance of the CSC population [17]. In this report, we found that Ixabepilone treatment increased autophagy with a concomitant increase in EGFR signaling, while Cetuximab inhibited autophagy by reducing the expression of the autophagy markers p62 and LC3b. Therefore, we speculate that the inhibition of autophagy by Cetuximab may have played a role in reducing the CSC population. However, Li et al. have also shown that Cetuximab induces autophagy in A431 human vulvar squamous carcinoma cells, DiFi colorectal adenocarcinoma cancer cells, and HCC827 human non-small cell lung cancer cells by downregulating HIF-1α and Bcl-2 and activating the beclin-1/hVps34 complex [32]. Thus, the regulation of autophagy by Cetuximab may differ depending on the type of cancer being studied.

## Conclusions

These studies show that EGFR-targeted therapy effectively reduces CSC populations in SUM159 and MDA-MB-231 tumors and tumor growth in vivo. However, our in vivo data suggest that the effects of the combination therapy of Cetuximab and Ixabepilone may vary based on the TNBC subtype and support recent clinical trial results. Thus, EGFR-targeted therapy should be considered with caution for TNBC patients, and further investigation of TNBC subtypes, using patient-derived xenograft TNBC models [33, 34], may help to determine TNBC patient groups suitable for the combination therapy.

## Additional file

Additional file 1: Figure S1. Cetuximab treatment reduces autophagy. Western blot showed effects of cetuximab, ixabepilone, and combination (cetuximab + ixabepilone) treatment in MDA-MB-231 and SUM159 cells. Immunoblots were probed with antibodies against pEGFR, total EGFR, pARK, total ARK, p62, LC3b, pERK1/2, total ERK1/2, and b-Actin. Protein expression has been quantitated and normalized against the loading control, and the results of median of three replicates are represented in the table. (TIF 7 MB)

## Abbreviations

ALDF: Aldefluor
ALDH: aldehyde dehydrogenase
ANOVA: analysis of variance
CSC: cancer stem cell
DCIS: ductal carcinoma in situ
DMEM: Dulbecco’s modified Eagle’s medium
EGFR: epidermal growth factor receptor
ER: estrogen receptor
ERK: extracellular signal-regulated kinase
FACS: fluorescence-activated cell sorting
HBSS: Hank’s balanced salt solution
HER2: human epidermal growth factor receptor 2
i.p.: intraperitoneal
i.v.: intravenous
MBC: metastatic breast cancer
MEGM: mammary epithelial growth medium
MSFE: mammosphere formation efficiency
PBS: phosphate-buffered saline
PR: progesterone receptor
TDT: tumor doubling time
TNBC: triple-negative breast cancer
