# Supplementary figures and images for: Antitumor activity of Cetuximab in combination with Ixabepilone on triple negative breast cancer stem cells
Source: Breast Cancer Res. 2016 Jan 12;18:6. doi: 10.1186/s13058-015-0662-4 (PMC4711100; doi:10.1186/s13058-015-0662-4)

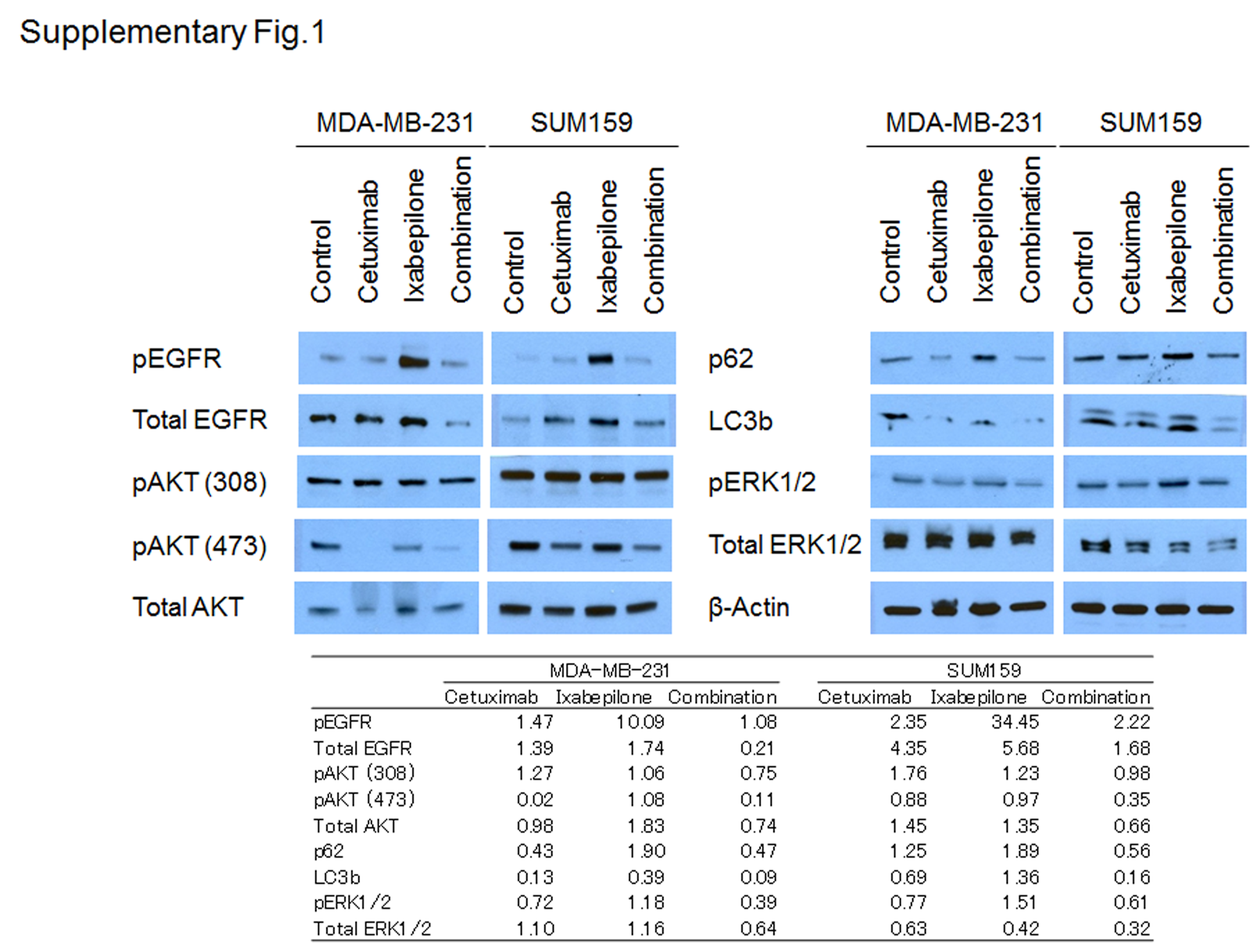

Supplement: Additional file 1: Figure S1. — Cetuximab treatment reduces autophagy. Western blot showed effects of cetuximab, ixabepilone, and combination (cetuximab + ixabepilone) treatment in MDA-MB-231 and SUM159 cells. Immunoblots were probed with antibodies against pEGFR, total EGFR, pARK, total ARK, p62, LC3b, pERK1/2, total ERK1/2, and b-Actin. Protein expression has been quantitated and normalized against the loading control, and the results of median of three replicates are represented in the table. (TIF 7 MB) [file 13058_2015_662_MOESM1_ESM.tif]
